# Supplementary material for: Contribution of Intrinsic Fluorescence to the Design of a New 3D-Printed Implant for Releasing SDABS
Source: Pharmaceutics. 2020 Sep 26;12(10):921. doi: 10.3390/pharmaceutics12100921 (PMC7601711; doi:10.3390/pharmaceutics12100921)
Supplement: Supplementary file 1 [file pharmaceutics-12-00921-s001.pdf]

pharmaceutics

MDPI

Supplementary Materials: Contribution of Intrinsic Fluorescence to the Design of a New 3D-Printed Implant for Releasing SDABS

Alexandre Nicolas, Alice Dejoux, Cécile Poirier, Nicolas Aubrey, Jean-Manuel Péan and Florence Velge-Roussel

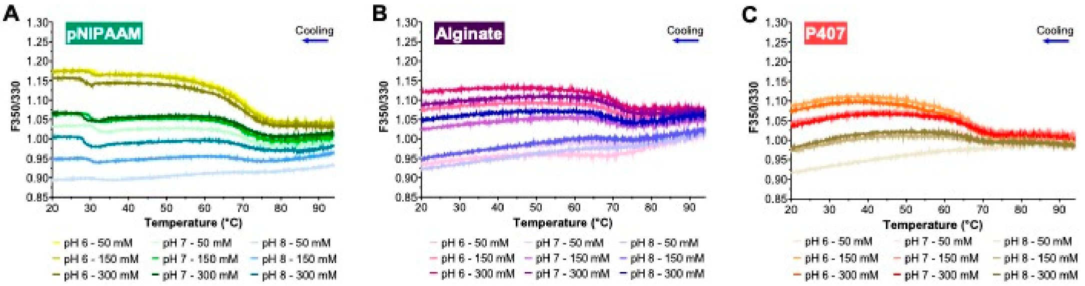

Figure S1. VHHHER2 thermal stability refolding in the presence of polymer. Wavelength intensity fluorescence 350/330 ration in the presence of pNIPAAM (**A**), alginate (**B**) and P407 (**C**) at different pH and ionic strength conditions during cooling ramp (color legend in the figure).

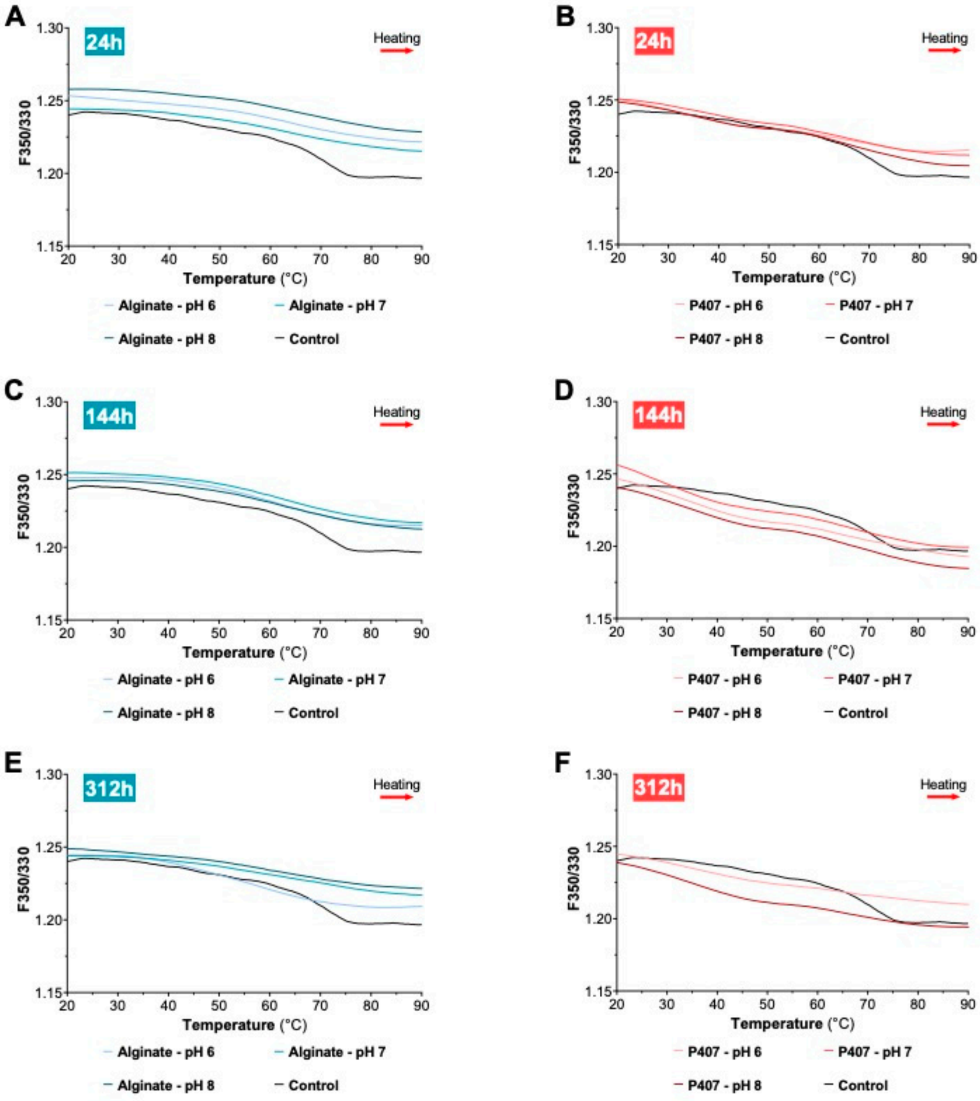

Figure S2. VHH$_{HER2}$ thermal stability after its release from alginate and P407 hydrogels. Wavelength intensity fluorescence 350/330 ration of VHH$_{HER2}$ in the release medium following its release from alginate (blue) and P407 (red) at 24 h (**A** and **B**), 144 h (**C** and **D**) and 312 h (**E** and **F**).
